# Identification of SHCBP1 as a novel downstream target gene of SS18-SSX1 and its functional analysis in progression of synovial sarcoma

**DOI:** 10.18632/oncotarget.11651

**Published:** 2016-08-27

**Authors:** Changliang Peng, Hui Zhao, Wei Chen, Yan Song, Xiaoying Wang, Ji Li, Yong Qiao, Dongjin Wu, Shengzhong Ma, Xiuwen Wang, Chunzheng Gao

**Affiliations:** ^1^ Department of Orthopaedics, Shandong University Second Hospital, Jinan, China; ^2^ Department of Orthopaedics, Beijing Chaoyang Hospital, Capital Medical University, Beijing, China; ^3^ Beijing Institute of Pharmacology and Toxicology, Beijing, China; ^4^ Nephrology Research Institute, Shandong University Second Hospital, Jinan, China; ^5^ Department of Pathology, Shandong University Second Hospital, Jinan, China

**Keywords:** synovial sarcoma, SS18-SSX1, SHCBP1, proliferation, apoptosis

## Abstract

The SS18-SSX1 fusion gene has been shown to play important roles in the development of synovial sarcoma (SS), but the underlying molecular mechanisms and its downstream target genes are still not clear. Here SHC SH2-domain binding protein 1 (SHCBP1) was identified and validated to be a novel downstream target gene of SS18-SSX1 by using microarray assay, quantitative real-time (qPCR) and western blot. Expression of SHCBP1 was firstly confirmed in SS cell line and SS tissues. The effects of SHCBP1 overexpression or knockdown on SS cell proliferation and tumorigenicity were then studied by cell proliferation, DNA replication, colony formation, flow cytometric assays, and its *in vivo* tumorigenesis was determined in the nude mice. Meanwhile, the related signaling pathways of SHCBP1 were also examined in SS cells. The results indicated that SHCBP1 was significantly increased in SS cells and SS tissues compared with adjacent noncancerous tissues. The expression of SHCBP1 was demonstrated to be positively correlated with the SS18-SSX1 level. Overexpression and ablation of SHCBP1 promoted and inhibited, respectively, the proliferation and tumorigenicity of SS cells *in vitro*. SHCBP1 knockdown also significantly inhibited SS cell growth in nude mice, and lowered the MAPK/ERK and PI3K/AKT/mTOR signaling pathways and cyclin D1 expression. Our findings disclose that SHCBP1 is a novel downstream target gene of SS18-SSX1, and demonstrate that the oncogene SS18-SSX1 promotes tumorigenesis by increasing the expression of SHCBP1, which normally acts as a tumor promoting factor.

## INTRODUCTION

As an aggressive tumor of soft tissue, synovial sarcoma (SS) represents approximately ten percent in total soft tissue sarcomas, which affect predominantly children and young adults [1]. Monophasic and biphasic forms are the two major histological types of SS. Despite recent advances in therapies, the 5-year survival rate for SS remains only 36% and less than 10% for patients with metastasis [2, 3].

Reciprocal t(X; 18) translocation that results in formation of a fusion protein product SS18-SSX (1–4) is a characterization of SS. This chromosomal translocation usually originates from the fusion of the SS18 gene that locates on chromosome 18 p11 to the SSX1 or SSX2, or occasionally the SSX4 gene that locates on chromosome Xq11 [4, 5]. This SS18-SSX fusion is specifically expressed in more than 95% of cases [6]. Currently, molecular detection the transcripts of the SS18-SSX fusion represents the most specific and sensitive diagnostic method for SS [7, 8]. Although we knew little about the function of the SS18-SSX fusion, it was confirmed to be responsible for cell growth of the SS [9, 10]. And the type of SS18-SSX fusion transcript was also correlated with the clinical behavior of SS [11, 12]. Tumors with SS18-SSX1 fusion transcript present worse prognosis than those with SS18-SSX2 [12, 13]. Our previous preliminary study suggested that the growth of SS cells could be significantly inhibited after SS18-SSX1 gene knockdown [14]. Takenaka S et al. also reported similar findings [15]. However, the molecular mechanisms underlying the coordinated regulation of SS18-SSX1 downstream target genes are largely unknown. To address this, the global gene expression patterns in SS cells after silencing SS18-SSX1 gene by RNAi were specially analyzed and compared by DNA microarray analysis. The comprehensive gene expression profile after SS18-SSX1 depletion revealed total 833 genes (including 510 upregulated and 323 downregulated genes) as possible mediators of SS18-SSX1 tumorigenic effects. Among them, the SHCBP1 gene was found to be a new downstream target of SS18-SSX1. As an evolutionarily conserved and ubiquitously expressed protein, SHCBP1 couples activated growth factor receptors-related signaling pathways [16], and plays important roles in cell growth and proliferation, differentiation, early embryonic survival and development, growth factor signaling pathway, and especially carcinogenesis [17–20]. SHCBP1, a new signaling pathway downstream of SHC adaptor proteins, is frequently found to be upregulated in several human malignancies including breast cancer, hepatocellular carcinoma, and certain leukemia/lymphoma [19–23]. Considering the relationship between SHCBP1 and SS18-SSX1, it is of great interest to investigate SHCBP1 expression and its biological function in SS.

For the first time, we confirmed the expression of SHCBP1 in SS cell line and SS specimens, and then the effects of SHCBP1 overexpression or knockdown on cell proliferation and tumorigenicity were further assessed in both vitro and vivo. SHCBP1 expression was found remarkably increased in SS cells and SS tissues. Furthermore, we showed that SHCBP1 can promote proliferation and tumorigenicity of SS cells in both vitro and vivo. Moreover, activation of the MAPK/ERK and PI3K/AKT/mTOR signaling pathways was demonstrated to be mechanistically related to the biologic behavior of SHCBP1. Our findings disclose that SHCBP1 is a novel downstream target gene of SS18-SSX1, and demonstrate that the oncogene SS18-SSX1 promotes tumorigenesis by enhancing the levels of SHCBP1, that normally serves as a cancer promoting factor.

## RESULTS

### Identification of SHCBP1 as a novel downstream target gene of SS18-SSX1

**Figure 1:**
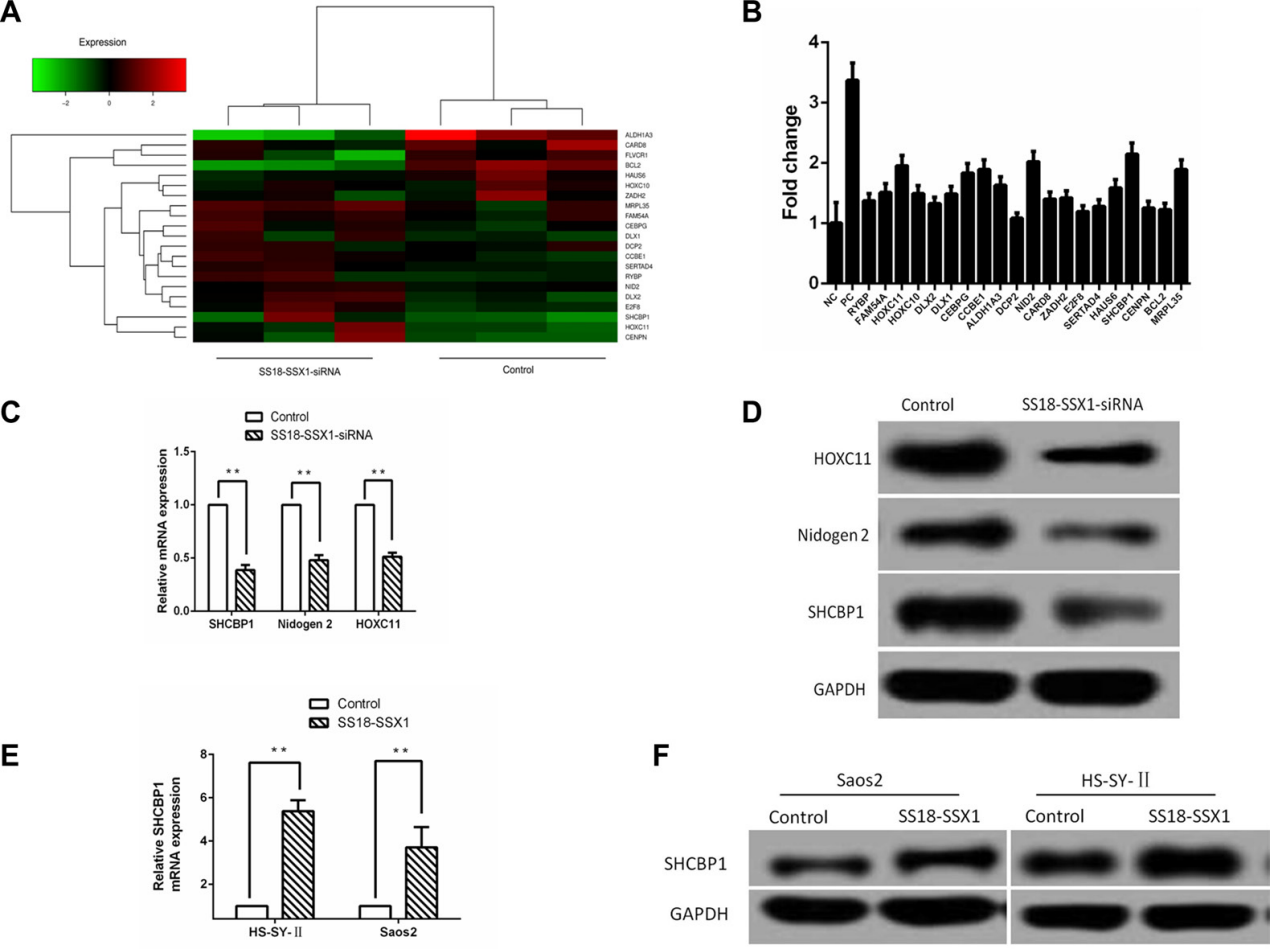
Identification of SHCBP1 as a SS18-SSX1 target gene (**A**) The effects of SS18-SSX1-siRNA on expression of target genes. Heat map represents the expression of SS18-SSX1 regulated genes. (**B**) Effect of downregulated 20 candidate downstream target genes of SS18-SSX1 on SS cells growth at 5th day. Cell growth was assayed by HCS assay every day for 5 days. Fold change was calculated by comparing the cell numbers of NC lentivirus infected cells to the siRNA lentivirus infected cells at 5th day. (**C**) qPCR validation of the genes downregulated by SS18-SSX1-siRNA in HS-SY-II cells (*n* = 3), and expressed relative to GAPDH expression. (**D**) Lysates were prepared from SS18-SSX1-siRNA cells and immunoblotted as indicated. (**E** and **F**) Elevated gene and protein expressions of SHCBP1 in SS18-SSX1-overexpressing Saos-2 and HS-SY-II cells measured by qPCR (E) and western blot analysis (F). **p < 0.01.

To identify potential SS18-SSX1 targets, we performed a microarray profiling (Affymetrix) in human SS cell line HS-SY-II cells infected with the recombinant lentivirus targeting SS18-SSX1. The lentiviral transduction efficiency was determined by fluorescence microscope through monitoring GFP expression at 72 h after transduction (Supplementary Figure S1A). The efficiency of SS18-SSX1 knockdown by the SS18-SSX1-siRNA was confirmed by qPCR (Supplementary Figure S1C) and western blotting (Supplementary Figure S1E). Compared with NC-siRNA as the control, the expression levels of 833 genes were significantly altered following SS18-SSX1 RNAi. Among these differentially expressed genes, 323 were downregulated, and 510 were upregulated. Of the downregulated genes, 20 displayed at least five-fold or more changes in expression level. The 20 identified SS18-SSX1 target genes included the following: SHCBP1, NID2, HOXC11, MRPL35, CCBE1, CEBPG, ALDH1A3, HAUS6, FAM54A, HOXC10, DLX1, ZADH2, CARD8, RYBP, DLX2, SERTAD4, CENPN, BCL2, E2F8, and DCP2 (Table 1 and Figure 1A).

**Table 1:** Twenty candidate downstream target genes of SS18-SSX1 identified by microarray assay were analyzed by RNAi

Gene symbol	GenBank_ID	Description	Target Sequence
NC		Non-targeting shRNA	
PC	NM_002592	proliferating cell nuclear antigen shRNA	
SHCBP1	NM_024745	SHC SH2 domain-binding protein 1	CTTGGTGAAACCTACAATCTT
NID2	NM_007361	nidogen 2 (osteonidogen)	CCGGCATACTTGCATCTTGAT
HOXC11	NM_014212	homeobox C11	CCCTTATTCGAAATTCCAGAT
MRPL35	NM_016622	mitochondrial ribosomal protein L35	GAGGAGAAAGGCTGGCTATAA
CCBE1	NM_133459	collagen and calcium binding EGF domains 1	GCTACTTATGCTGGCTGACAT
CEBPG	NM_001806	CCAAT/enhancer binding protein (C/EBP), gamma	CTGACCAAGGAATTAAGTGTA
ALDH1A3	NM_000693	aldehyde dehydrogenase 1 family, member A3	GCCGAATACACAGAAGTGAAA
HAUS6	NM_017645	HAUS augmin-like complex, subunit 6	GCACATAAGCAACATAACCAA
FAM54A	NM_001099286	mitochondrial fission regulator 2	CCAAACATGTTGGACGTTCTA
HOXC10	NM_017409	homeobox C10	CTGGAGATTAGCAAGACCATT
DLX1	NM_178120	distal-less homeobox 1	CCCTAACTCTTCATCCGGGAA
ZADH2	NM_175907	zinc binding alcohol dehydrogenase domain containing 2	CGCTTGATAGTAATAGGGTTT
CARD8	NM_001184900	caspase recruitment domain family, member 8	CCTATCTTAGACAGCAGAATT
RYBP	NM_012234	RING1 and YY1 binding protein	CACCGTCATTATCACAGACTT
DLX2	NM_004405	distal-less homeobox 2	GCCTGAAATTCGGATAGTGAA
SERTAD4	NM_019605	SERTA domain containing 4	CTGTCAATGCTAATGTTGGAA
CENPN	NM_018455	centromere protein N	TGAACTGACAACAATCCTGAA
BCL2	NM_000633	B-cell CLL/lymphoma 2	CGCCCTGTGGATGACTGAGTA
E2F8	NM_024680	E2F transcription factor 8	GCCCAGAAATCAGTCCAAATA
DCP2	NM_152624	decapping mRNA 2	CCGTGGCATGTAATGGACATT

Notes: NC: Non-targeting shRNA (negative control); PC: proliferating cell nuclear antigen (PCNA) shRNA (positive control).

To assess whether the expressions of 20 selected genes from the above results are important for the proliferation or survival of SS cells, we first constructed 20 lentivirus-mediated siRNAs (Table 1) to knock down the expression of the above 20 genes in the HS-SY-II cells. GFP-expressing cells were calculated for five continuous days by high content screening (HCS) assay after infection with the lentivirus containing the above mentioned 20 genes siRNAs or NC-siRNA. As shown in Figure 1B, we observed HS-SY-II cells with GFP in 5 days and assessed the cell proliferation at 5th day with fold changes of the targeted siRNA-treated groups relative to the negative control group. Those genes whose fold change was over 1.9 and the *p-value* was lower than 0.001 were considered to be differently expressed. Our results indicated that the growth of cells was significantly inhibited by interfering SHCBP1 (2.14-fold change), NID2 (2.02-fold change) and HOXC11 (1.95-fold change) (*p* < 0.001), respectively (Figure 1B). Among the 20 downstream target genes of SS18-SSX1, SHCBP1 was identified to be one of the most significant.

To determine whether the expression of these genes was indeed decreased by SS18-SSX1-siRNA, gene expression was assayed by qPCR. We found the expression of SHCBP1, NID2 and HOXC11 was decreased in SS18-SSX1-siRNA cells (Figure 1C). Similar results were obtained when we performed immunoblotting for these proteins (Figure 1D).

To directly confirm the relationship between SS18-SSX1 expression and SHCBP1 levels, we overexpressed SS18-SSX1 in Saos-2 (not expressing endogenous SS18-SSX1) and HS-SY-II cells by plasmid-mediated transduction. The transfection efficiency was confirmed by qPCR (Supplementary Figure S1B) and western blotting (Supplementary Figure S1D). SS18-SSX1 overexpression resulted in markedly higher levels of SHCBP1 (Figure 1E). Similarly, immunostaining of SS18-SSX1-overexpressing cells showed enhanced SHCBP1 expression (Figure 1F). These results show that SHCBP1 is a novel SS18-SSX1 target gene.

### SHCBP1 was overexpressed in SS

**Figure 2:**
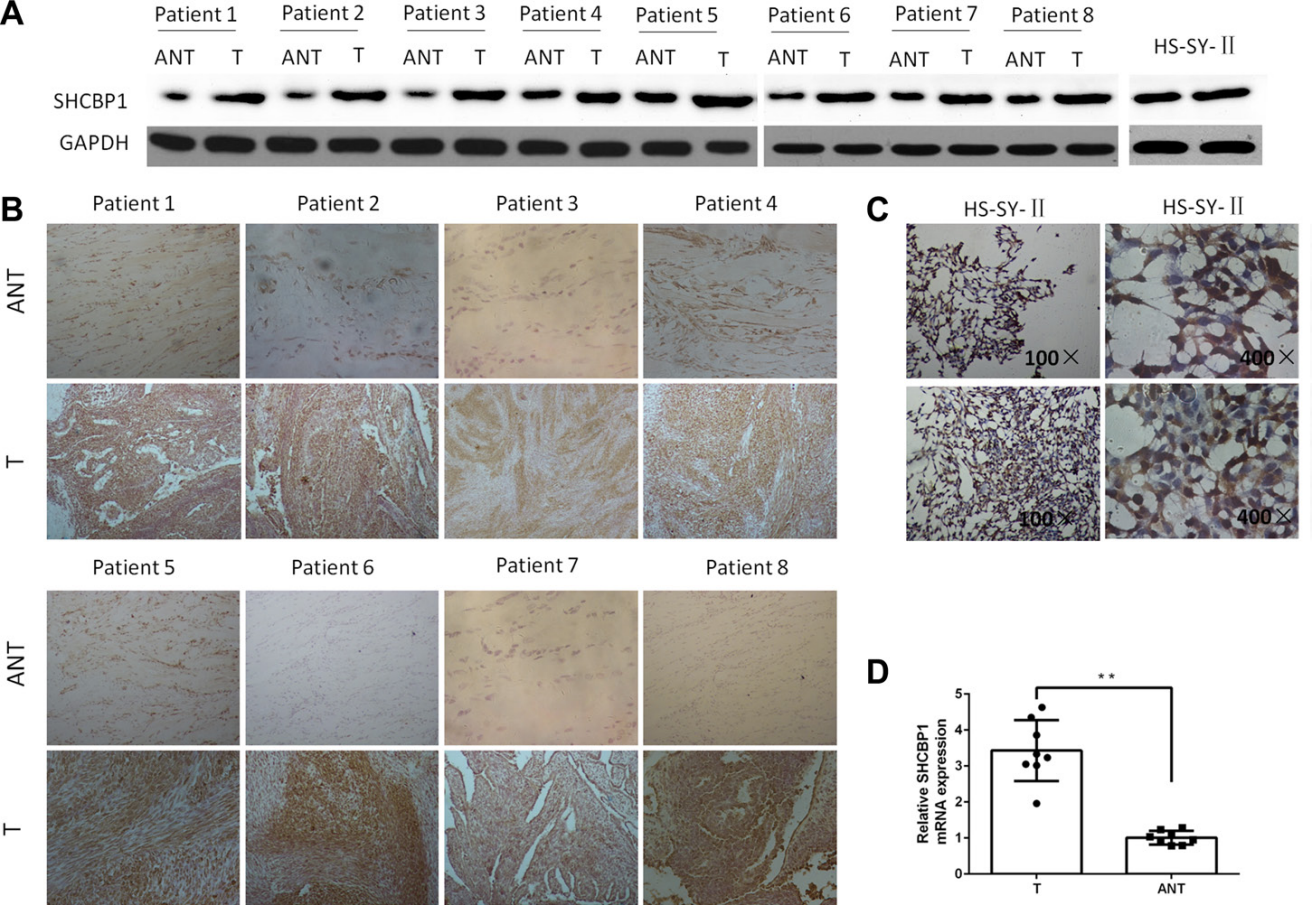
SHCBP1 expression is overexpressed in SS cell line and SS tissues (**A** and **B**) Western blot and IHC examination of SHCBP1 expression in matched SS tissues (T) and adjacent noncancerous tissues (ANT) and HS-SY-II cells. GAPDH served as the loading control in the western blot assay. IHC shows strong immunoreactivity of SHCBP1 in the SS tissues (T), while weak immunoreactivity of SHCBP1 in adjacent noncancerous tissues (ANT). (**C**) ICC shows strong immunoreactivity of SHCBP1 in the cytoplasm of HS-SY-II cells. (**D**) The higher expression level of SHCBP1 mRNA in SS tissues (T) versus the adjacent noncancerous tissues (ANT). SHCBP1 mRNA expression was detected by qPCR, and normalized to that of GAPDH. **p < 0.01.

We first assessed the SHCBP1 gene expression in eight matched SS tissues and adjacent noncancerous tissues using qPCR, western blot analysis and immunohistochemistry (IHC). The results revealed that, when comparing with the adjacent noncancerous tissues, the relative mRNA and protein expression levels of SHCBP1 were markedly increased in SS tissues (Figure 2A and 2D). Furthermore, expression of SHCBP1 protein was also detected in eight matched SS tissues and adjacent noncancerous tissues by IHC (Figure 2B). IHC analysis showed that the adjacent noncancerous tissues showed low levels of SHCBP1 staining, in contrast to SS, which exhibited strong SHCBP1 staining (Figure 2B). The staining results showed that SHCBP1 protein is mainly located in the cytoplasm in SS cells (Figure 2B). Moreover, we further confirmed the gene and protein expression of SHCBP1 in HS-SY-II cell line by qPCR (the average Ct value of GAPDH and SHCBP1 is 14.99 and 24.24, respectively) and immunocytochemistry (ICC) (Figure 2C), respectively.

### The impact of overexpression or knockdown of SHCBP1 on SS cell growth at an *in vitro* level

**Figure 3:**
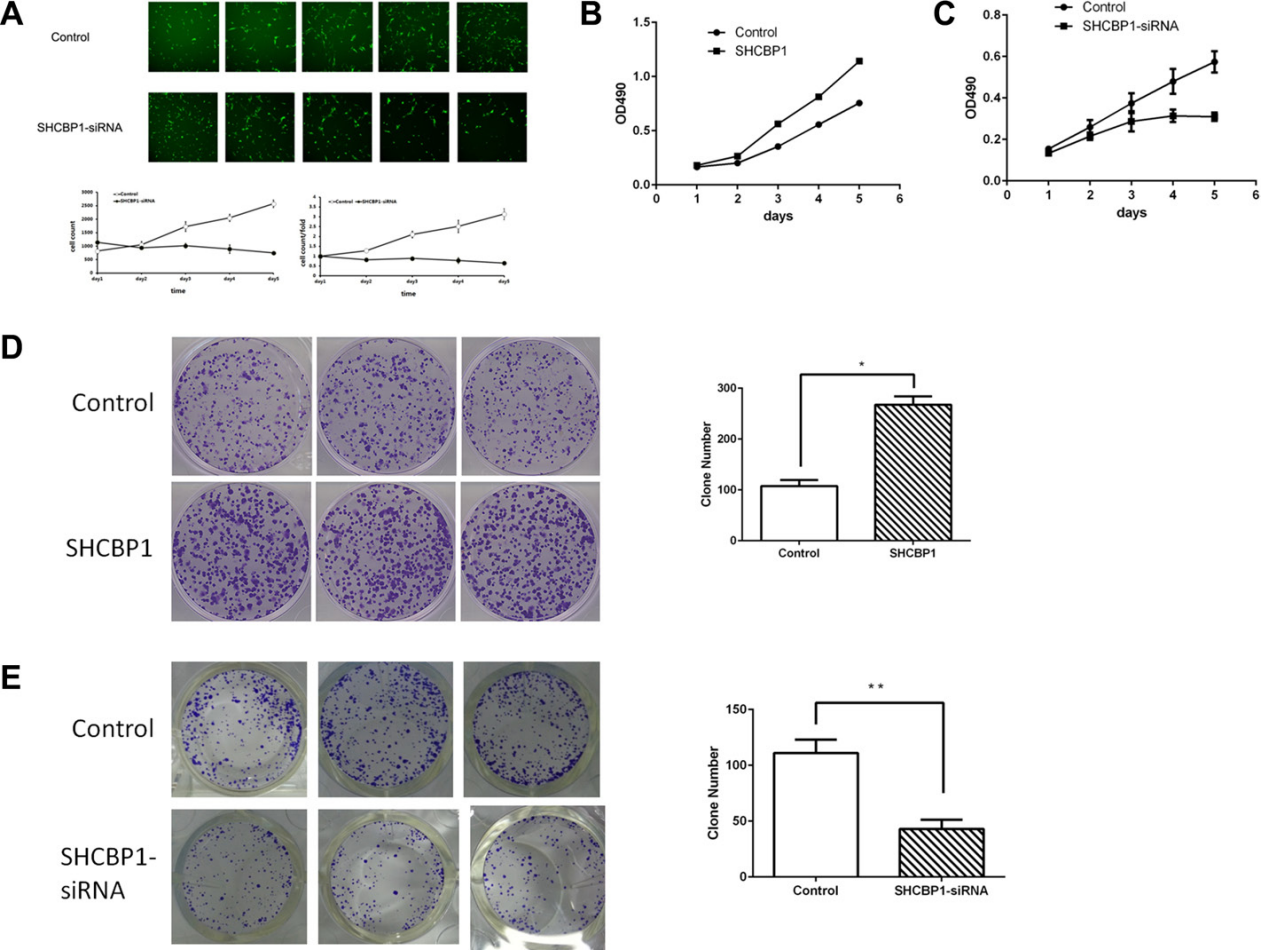
Effect of SHCBP1 on SS cells growth *in vitro* (**A**) The cell's growth rates were determined by HCS assay at indicated time points. MTT assays (**B** and **C**) and colony formation assay (**D** and **E**) indicated increased growth rates in SHCBP1-overexpressing cells (B and D) and decreased growth rates in SHCBP1-silenced cells (C and E). Values are mean ± SD of 3 independent experiments; *p < 0.05, **p < 0.01.

To further determine whether SHCBP1 affects the proliferation, HS-SY-II cells stably overexpressing SHCBP1 were established. The transfection efficiency was confirmed by qPCR (Supplementary Figure S2B) and western blotting (Supplementary Figure S2D). Then we performed *in vitro* MTT and colony formation assays. As shown in Figure 3B, the proliferation rate was significantly increased in SHCBP1-overexpressing HS-SY-II cells, as compared with control cells. These results were further confirmed by colony formation assay, and as shown in Figure 3D, SHCBP1-overexpressing cells displayed obviously more numerous and larger colonies compared with control cells.

To determine how SHCBP1 promotes cell viability, SHCBP1 expression was silenced using lentivirus-mediated RNA interference technology. The transfection efficacy of the lentiviral vectors was viewed by checking GFP expression under a fluorescence microscope 72 h post-transfection (Supplementary Figure S2A). The RNAi efficiency was confirmed by qPCR (Supplementary Figure S2C) and western blotting (Supplementary Figure S2E). The promoting effects of SHCBP1 on proliferation of SS cells were assessed by HCS, MTT and colony formation assays. As shown in Figure 3A and 3C, compared with NC-siRNA lentivirus infected cells, the growth of SHCBP1-siRNA lentivirus infected cells was significantly suppressed. On day 5, OD_490_ of SHCBP1-siRNA lentivirus infected cell was only 0.309 ± 0.02, while that of NC-siRNA lentivirus infected cells was 0.574 ± 0.052 (Figure 3C). We then analyzed the effect of SHCBP1 on the clonogenicity of SS cells. As shown in Figure 3E, the size and the number of colonies in the SHCBP1-siRNA lentivirus infected cells were significantly reduced compared to NC-siRNA lentivirus infected cells. Collectively, our results suggest that SHCBP1 promotes the proliferation and tumorigenicity of SS cells *in vitro*.

### SHCBP1 promotes the transition from G1 to S phase in SS cells

**Figure 4:**
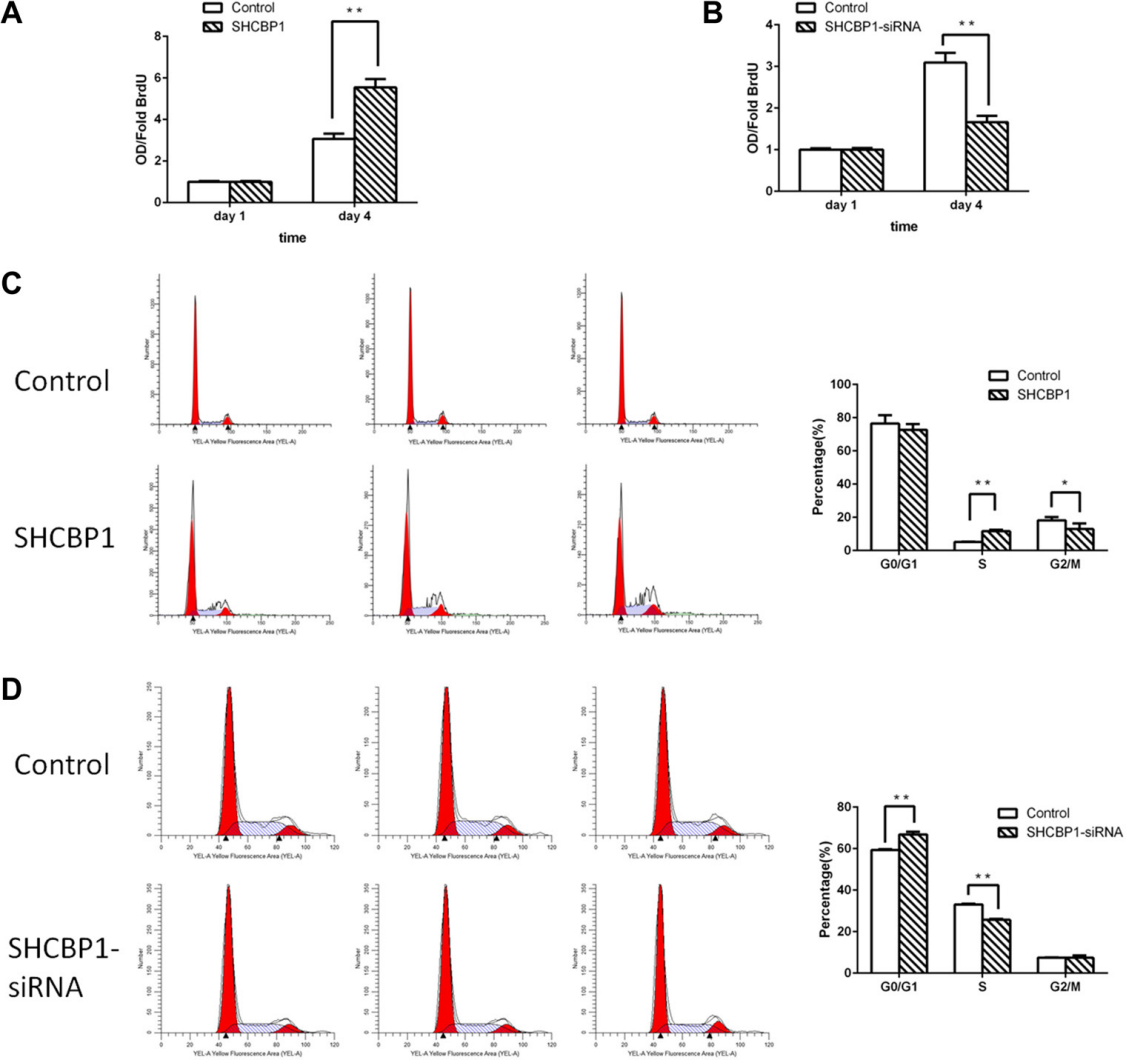
SHCBP1 promotes the transition from G1 to S phase in SS cells (**A** and **B**) BrdU incorporation assay was used to determine the DNA synthesis on the first and fourth day in SHCBP1-overexpressing (A) and SHCBP1-silenced (B) cells. (**C** and **D**) Flow cytometric analysis of cell cycle in SHCBP1-overexpressing (C) and SHCBP1-silenced (D) cells. All values are mean ± SD of 3 independent experiments; *p < 0.05, **p < 0.01.

To elucidate the mechanism of the promoting effects of SHCBP1 on the proliferation capacity of SS cells, the BrdU incorporation and flow cytometric assays were conducted. As shown in Figure 4A, overexpression of SHCBP1 in HS-SY-II cells markedly increased the percentage of BrdU incorporated cells, in contrast, knockdown of SHCBP1 significantly decreased that (Figure 4B). Accordingly, flow cytometry assay indicated the percentage of S-phase cells was significantly increased after overexpression of SHCBP1 and the percentage of G1/G0 phase cells was reduced (Figure 4C), whereas knockdown of SHCBP1 led to opposite results (Figure 4D). Taken together, the above results suggest SHCBP1 contributes to the transition from G1 to S phase in SS cells.

### Silencing of SHCBP1 induced apoptosis of HS-SY-II cells

Annexin V-APC staining by FACS on HS-SY-II cells following lentivirus infection was further utilized to confirm the influence of SHCBP1 on cell apoptosis. Figure 6B showed the apoptotic rate in SHCBP1-siRNA lentivirus infected cells was significantly higher than that of NC-siRNA lentivirus infected cells (37.99 ± 0.99% and 9.33 ± 0.46%, respectively), suggesting that SHCBP1 knockdown promoted apoptosis of the SS cells. In the following, the effect of SHCBP1 overexpression on apoptosis of SS cells was also determined by flow cytometry assay. As expected, no significant difference in cell apoptosis was identified between the SHCBP1-overexpressing and control cells (*p* > 0.05; Figure 6A). Altogether, SHCBP1 might play an oncogenic role in SS again.

### Silencing of SHCBP1 suppressed SS cell growth *in vivo*

**Figure 5:**
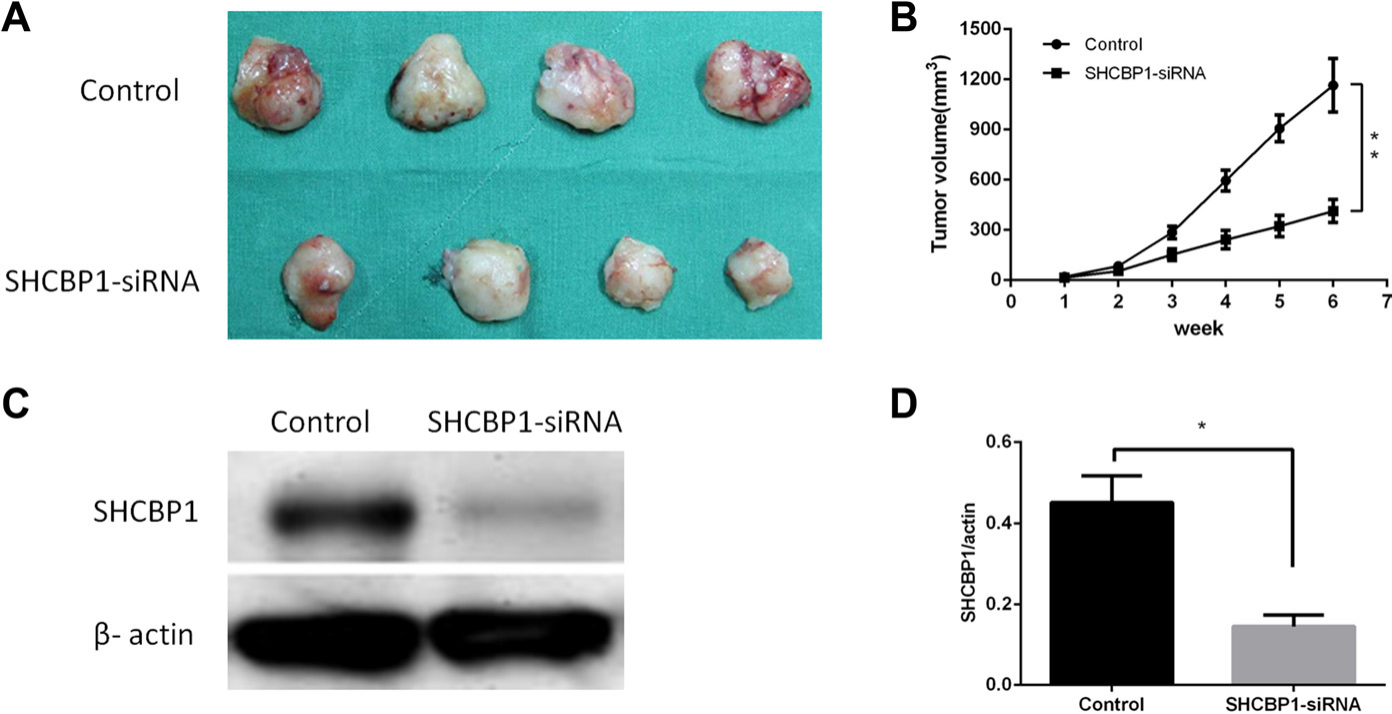
Inhibition of SHCBP1 reduces the tumorigenicity of SS cells *in vivo* (**A**) Photograph of xenografts dissected from nude mice after 6 weeks subcutaneous inoculation showing suppression growth of cancer cells transfected with SHCBP1-siRNA rather than control. (**B**) Tumor growth curve showing a significant growth tendency in control group as compared to the SHCBP1-siRNA transfected group. (**C**) SHCBP1-siRNA inhibited the protein expression of SHCBP1 *in vivo* as determined by western blot. (**D**) The bar represented that the relative expression of SHCBP1 in SHCBP1-siRNA group was significantly inhibited compared to that in control group; **p < 0.01.

To confirm whether silencing of SHCBP1 could inhibit the growth of SS cells *in vivo*, a subcutaneous human SS nude mouse xenograft model was established. The mouse group infected with SHCBP1-siRNA lentivirus had a lower proliferation rate, and formed evidently smaller tumors than the NC-siRNA lentivirus group, as shown in Figure 5A and 5B. The tumor size at the time of death in the SHCBP1-siRNA lentivirus group was 413 ± 69.6 mm^3^, which was significantly smaller than in the NC-siRNA group (1165 ± 160.3 mm^3^). In addition, a western blotting assay was performed to determine whether SHCBP1 expression was suppressed by SHCBP1-siRNA *in vivo*, and we found that the expression of SHCBP1 was largely inhibited by SHCBP1-siRNA (Figure 5C and 5D). These data suggest that SHCBP1-siRNA reduces tumor volume and growth rate of SS cells *in vivo*.

**Figure 6:**
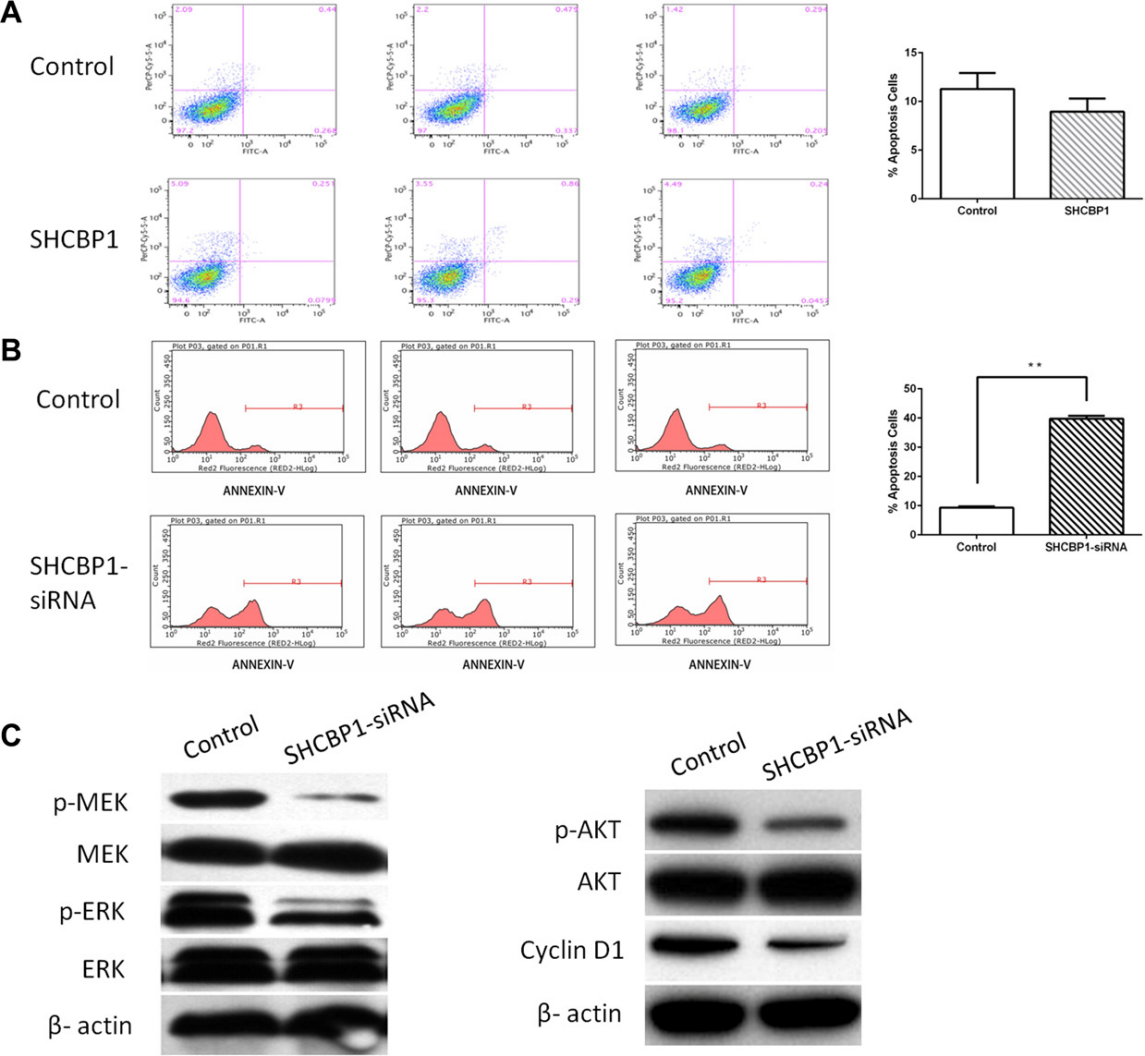
SHCBP1 exerts anti-apoptotic effect via activating MAPK/ERK and PI3K/AKT/mTOR signaling pathways and enhancing the expression of cyclin D1 A and B. Apoptosis was determined by flow cytometry in SHCBP1-overexpressing (**A**) and SHCBP1-silenced cells (**B**). Cells stained with annexin-V were considered as apoptosis. The apoptotic index was defined as the percentage of apoptotic cells. (**C**) The levels of phosphorylated MEK, total MEK, phosphorylated ERK, total ERK, phosphorylated Akt, total Akt, and cyclin D1 were detected in control and SHCBP1-siRNA cells by western blot analysis. β-actin was used as the loading control. Values are mean ± SD of 3 independent experiments; **p < 0.01.

### Silencing of SHCBP1 effectively promotes the inactivation of MAPK/ERK and PI3K/AKT/mTOR signaling pathways and reduces the expression of cyclin D1 in SS cells

SHC1, SHC (Src homology 2 domain containing) transforming protein 1, is important for normal and oncogenic signaling by epidermal growth factor receptor (EGFR) family receptor tyrosine kinases [24]. Phosphorylation of SHC1 by EGFR will stimulate the Mitogen-activated protein kinase (MAPK/ERK) and PI3K/AKT/mTOR signaling pathways [17], and control the growth of SS [25]. SHCBP1 binds with SHC1, which may be required for activation of MAPK/ERK and PI3K/AKT/mTOR signaling pathways. Therefore, the effects of silencing of SHCBP1 on MAPK/ERK and PI3K/AKT/mTOR signaling pathways in SS cells were assessed by western blotting. As shown in Figure 6C, the expression levels of the total MEK, ERK and AKT in HS-SY-II cells were unchanged, but the phosphorylations of these proteins were significantly decreased after silencing of SHCBP1. These data indicate that SHCBP1 silencing effectively promotes the inactivation of MAPK/ERK and PI3K/AKT/mTOR signaling pathways.

As an important regulator of cell cycle progression, cyclin D1's deregulation is linked to the pathogenesis of SS [9, 14]. To determine whether SHCBP1 silencing influences cyclin D1 expression, we detected the cyclin D1 level after SHCBP1 silencing by western blot analysis. As indicated in Figure 6C, knocking down SHCBP1 could significantly reduce the expression of cyclin D1.

## DISCUSSION

Recently accumulating data showed that SS18-SSX plays critical roles in oncogenesis and development of SS [26, 27]. Our previous study indicated that inhibition of SS18-SSX by siRNA could prevent the proliferation of SS cells [14]. However, the downstream molecular mechanisms involved in SS18-SSX1-mediated oncogenesis are still poorly understood.

To identify new downstream targets of SS18-SSX1, a cancer-related gene expression panel with samples from SS18-SSX1-siRNA SS cells and control cells was carried out in our research. Twenty target genes including the following: SHCBP1, NID2, HOXC11, MRPL35, CCBE1, CEBPG, ALDH1A3, HAUS6, FAM54A, HOXC10, DLX1, ZADH2, CARD8, RYBP, DLX2, SERTAD4, CENPN, BCL2, E2F8, and DCP2 were identified. To investigate which of them is associated with cellular proliferation. The effect of 20 target genes knockdown on cell proliferation was firstly assessed by HCS assay. Three genes, including SHCBP1, NID2, and HOXC11, were identified as SS18-SSX1 downstream target genes. Individual siRNA-mediated knockdown of them was sufficient to inhibit the proliferation of SS cells. Among them, SHCBP1 which demonstrated the most significant fold changes (2.14-fold change) was identified as one of the most significantly target gene, whose expression was upregulated by SS18-SSX1 overexpression, and downregulated by SS18-SSX1 inhibition. To our knowledge, this is the first report that SHCBP1 is a downstream target gene of SS18-SSX1.

Three overlapping proteins encoded by the SHC gene share a common carboxy terminal SH2 domain [28–30], which plays an important role in the signal transduction pathways. Studies have also shown that SHC proteins mediate cell proliferation as well as cell survival via tyrosine phosphorylation signal pathway [31, 32]. The direct interaction of SHCBP1 with the SH2 domain of SHC is independent of tyrosine phosphorylation. SHCBP1, located on chromosome 16q11.2, also modulated the fibroblast growth factor signaling pathway in neural progenitor cells [33]. Studies have demonstrated that SHCBP1 is correlated with cell growth and proliferation, differentiation, early embryonic survival and development, growth factor signaling pathway, and especially carcinogenesis [17–20]. In breast cancer, SHCBP1 has been shown highly expressed in tumor samples and is correlated with metastatic potential, advanced stage, and poor prognosis [19]. Additionally, SHCBP1 was demonstrated significantly overexpressed in human hepatocellular carcinomas (HCC), and the proliferation and colony formation of HCC cells were markedly reduced after SHCBP1 inhibition [20]. Although SHCBP1 is indicated to be overexpressed in several kind of cancers, it has not been associated with SS. Here we found the expression of SHCBP1 is higher in SS cell line HS-SY-II. SHCBP1 gene and protein are significantly elevated in SS tissues compared with the adjacent noncancerous tissues. Meanwhile, in agreement with previous studies [17, 20], we found that staining of SHCBP1 is also mainly localized in the cytoplasm. These results indicated that SHCBP1 overexpression may take part in the oncogenic process of SS. The relationship between SHCBP1 expression and the clinical features of SS is definitely worth further exploration.

Functional investigations were mainly focused on the impact of overexpression or knockdown of SHCBP1 on the growth and apoptosis of SS cells. The results disclosed that cell proliferation, colony formation and DNA replication could be promoted in SHCBP1 overexpressed SS cells. In contrast, targeting SHCBP1 produced the opposite effects. Moreover, silencing of SHCBP1 led to remarkable apoptosis of HS-SY-II cells. Most importantly, the *in vivo* data demonstrated that silencing of SHCBP1 could significantly prohibit xenograft tumor growth in mouse model. These findings indicate that SHCBP1 is involved in carcinogenesis of SS, and thus it may be considered as one of the novel potential therapeutic targets in SS treatment. These findings reported here are consistent with a previous report in HCC cells [20].

Flow cytometry also showed that overexpression of SHCBP1 accelerated the G1-S-phase transition, whereas silencing of SHCBP1 induced G1-S-phase arrest. In addition, we found that silencing of SHCBP1 effectively inhibited the expression of cyclin D1 in SS cells. Thus, we showed that the mechanism of SHCBP1-mediated proliferation was linked to alternations of the expression of cyclin D1. Taken together, these results reveal that SHCBP1 involves in cell cycle regulation of SS and is critical for expression of cyclin D1. These findings are in accordance with previous reports [20, 34].

To investigate the underlying mechanisms by which SHCBP1 promotes proliferation of SS cells, we explored the effects of SHCBP1 silencing on the activity of MAPK/ERK and PI3K/AKT/mTOR signaling pathways which are important in the pathogenesis of various tumors [35–37] including SS [38, 39]. We speculated that SHCBP1 may cross talk with the MAPK/ERK and PI3K/AKT/mTOR signaling pathways in SS progression. The MAPK/ERK and PI3K/AKT/mTOR signaling pathways may be first stimulated by the elevated expression of SHCBP1, which leads to increased tumor growth potential. Our present data showed that the MAPK/ERK and PI3K/AKT/mTOR signaling pathways were inactivated by silencing of SHCBP1 in HS-SY-II cells via reducing the levels of phosphorylated MEK, ERK and AKT. Therefore, the result explains, at least in part, why SHCBP1 silencing inhibited the cell proliferation and induced the apoptosis in HS-SY-II cells, which is consistent with these reports [20, 33]. Molecular mechanism underlying SHCBP1 inactivating the MAPK/ERK and PI3K/AKT/mTOR signaling pathways in SS cells is under investigation in our laboratory currently.

Collectively, SHCBP1 was identified as a novel downstream target gene of SS18-SSX1 for the first time. SS18-SSX1 functions as an oncoprotein by promoting tumorigenesis via increasing the expression of SHCBP1 in SS, and then activating the MAPK/ERK and PI3K/AKT/mTOR signaling pathways. The precise underlying mechanism through which SS18-SSX1 increases the expression of SHCBP1 is still underway in our lab.

## MATERIALS AND METHODS

### Ethics statement

Investigation has been conducted in accordance with the ethical standards and according to the Declaration of Helsinki and according to national and international guidelines and has been approved by the institutional review board of the Second Hospital of Shandong University. For tissue specimens, patients were informed that the resected specimens were stored by the hospital and potentially used for scientific research, and that their privacy would be maintained. Informed consent has been obtained. For animal research, all experimental procedures were conducted in accordance with the Guide for the Care and Use of Laboratory Animals and approved by our institutional ethical guidelines for animal experiments.

### Tissue specimens and cell lines

Eight matched SS and adjacent noncancerous tissues were collected and were fixed with 10% neutral-buffered formalin and embedded in paraffin; 4 μm-thick sections were prepared for IHC. All cases of SS and adjacent noncancerous tissues were diagnosed clinically and pathologically.

The human SS cell line HS-SY-II, expressing the SS18-SSX1 transcript [40] was kindly provided by Professor Yi Guo (University of California, Irvine, USA). Human osteosarcoma cell line Saos-2 was obtained from the American Type Culture Collection (ATCC, Manassas, VA, USA). HS-SY-II cells were routinely cultured in Dulbecco's modified Eagle's medium (DMEM, Gibco, NY, USA) supplemented with 10% fetal bovine serum (FBS, Gibco). Saos-2 cells were grown in McCoy's 5a Modified medium (Gibco, NY, USA) containing 15% FBS. Both cell lines were maintained at 37°C in a humidified atmosphere with 5% CO_2_.

### Lentivirus-mediated RNA interference

pGCL-GFP-Lentivirus was used to express siRNA targeting SS18-SSX1 gene (Genbank no. AB300354) and SHCBP1 gene (Genbank no. NM_024745). The siRNA sequences targeting SS18-SSX1 (SS18-SSX1-siRNA) were as follows: sense 5′-GTTAACCAAGGCAATCATA-3′ and antisense 5′-TATGATTGCCTTGGTTAAC-3′; The siRNA sequences targeting SHCBP1 (SHCBP1-siRNA) were as follows: sense 5′-TGGTGAAACCTACAATCTT-3′ and antisense 5′-AAGATTGTAGGTTTCACCA-3′. The nonsilencing siRNA (NC-siRNA) sequences used as a negative control were following: sense 5′- TTCTCCGAACGTGTCACGT-3′ and antisense 5′- ACGTGACACGTTCGGAGAA-3′. siRNA constructs were synthesized and inserted into between the AgeI and EcoRI sites of pGCSIL-GFP plasmid vector (Genechem Co. Ltd., Shanghai, China) which contains the green fluorescent protein (GFP) gene as a reporter with an internal CMV promoter. Recombinant lentiviruses expressing SS18-SSX1-siRNA, SHCBP1-siRNA and nonsilencing siRNA were produced by Genechem (Shanghai, China). HS-SY-II cells were infected with recombinant lentivirus at a multiplicity of infection (MOI) of 50. Infection efficiency was measured by counting GFP-expressing cells under fluorescence microscope at 72 h after lentiviral infection. qPCR and western blot were then used to investigate the interference efficiency.

### Construction of plasmids and transfection

The SS18-SSX1 expression construct or SHCBP1 expression construct were generated by subcloning PCR-amplified full-length human SS18-SSX1 cDNA or full-length human SHCBP1 cDNA into the pcDNA.1(+) plasmid (Genechem), respectively. Cells were then transduced with the recombinant plasmid carrying the human SS18-SSX1 or SHCBP1 gene using Lipofectamine 2000 (Invitrogen) in accordance with the manufacturer's advised procedure. The effects of SS18-SSX1 overexpression on expression of SHCBP1 in Saos-2 cells or cultured HS-SY-II cells were determined by qPCR and western blot assays. Empty plasmid-transfected cells were used as control. Primer sequences for vectors construction are listed in Supplementary Table S1.

### Quantitative real-time PCR

Total RNA was purified with by Trizol reagent (Invitrogen, Carlsbad, CA, USA), and reversely transcripted to cDNA with M-MLV reverse transcriptase kit (Promega, USA) following the manufacturer's instructions. qPCR was performed with the SYBR Green Real-Time PCR assay kit (TAKARA, Otsu, Japan) on an ABI PRISM 7300 Sequence Detection System (Applied Biosystems, Foster City, California, USA). The 20 μl PCR reaction mixture was: 10 μl 2 × SYBR premix ex taq, 0.5 μl each primer (2.5 μM), 1 μl cDNA and 8 μl ddH_2_O. Cycling conditions were as follows: initial denaturation at 95°C for 15 s; denaturation 95°C for 5 s; annealing extension of 60°C for 30 s (a total of 45 cycles). The absorbance values were read at the extension stage. Fold changes in expression were calculated using the 2^−ΔΔCt^ method [41]. Experiments were performed at least three times. Primer sequences are listed in Supplementary Table S1.

### Immunohistochemistry and immunocytochemistry analysis

For IHC, tissue sections (4 μm) were placed on glass slides, heated at 70°C for 30 min, and then deparaffinized with xylene and ethanol. For antigen retrieval, the deparaffinized and rehydrated slides were heated in 10 mM citrate buffer for 20 min in a microwave oven and allowed to cool for 20 min at room temperature. Slides were incubated with 3% H_2_O_2_ in methanol for 15 min at room temperature to eliminate endogenous peroxidase activity. Then slides were incubated at 4°C overnight with antibody against the rabbit polyclonal anti-SHCBP1 antibody (1:50, Sigma-Aldrich, St Louis, MO, USA). After incubation with the biotinylated secondary antibody at room temperature for 20 min, the slides were incubated with a streptavidin-peroxidase complex at room temperature for 20 min. IHC staining was developed using 3,3′-diaminobenzidine (DAB) (Sigma-Aldrich) and counterstained with haematoxylin.

For ICC, cells were plated on 2.5 cm coverslips at a density of 5 × 10^5^ cells/well in 6-well plates. After 24 h of attaching, coverslips were fixed in 4% paraformaldehyde in PBS for 30 min and incubated with 3% H_2_O_2_ in methanol for 10 min. Coverslips were incubated with 10% goat serum at room temperature for 10 min to block non-specific bindings, and then immunostained with the anti-SHCBP1 antibody with the same protocol as IHC.

### Western blot analysis

Cells were harvested in RIPA buffer containing 100 mM Tris, 150 mM NaCl, 1 mM EDTA, 1% TritonX-100, 1% sodium deoxycholate, 0.1% SDS, PH 7.4, and supplemented with protease and phosphatase inhibitor cocktails (Sigma-Aldrich). Protein concentrations were determined by Bradford assay (Bio-Rad Laboratories, Hercules, CA, USA). Equal amounts of protein (30 μg) were electrophoresed by 10% SDS-PAGE and transferred onto a polyvinylidene fluoride (PVDF) membrane (Amersham, Piscataway, NJ, USA), and then the membrane was probed with the following antibodies: anti-SS18, anti-GAPDH, anti-HOXC11, anti-Nidogen 2, and anti-β-actin antibodies (Santa Cruz, 1:1000 dilution), anti-SHCBP1 polyclonal antibody (Sigma-Aldrich,1:2000 dilution), anti-p-MEK, anti-MEK, anti-p-ERK, anti-ERK, anti-p-AKT, anti-AKT, and anti-cyclin D1 antibodies (Cell Signaling Technology, 1 : 1000 dilution). Secondary antibodies conjugated to horseradish peroxidase and ECL Western blotting reagents were used for detection.

### Microarray assay

After 72 h infection with recombinant SS18-SSX1-siRNA lentivirus, total RNAs were extracted using the RNeasy Mini Kit (Qiagen, Basel, Switzerland). Then RNA quality was analyzed using an Agilent 2100 Bioanalyzer (Agilent, Santa Clara, CA, USA). Analysis of gene expression was performed using GeneChip^®^ PrimeView™ Human Gene Expression Array (Affymetrix, USA; catalog # 901838) which contains probes for 36000 genes as described previously [42]. Labeling and hybridization were performed following Affymetrix protocols. Primary array processing was conducted using the Affymetrix GeneChip^®^ Command Console^®^ Software (AGCC, version 1.1) and subsequent analysis was performed using the Affymetrix Expression Console (EC, version 1.1).

### High content screening assay

Cell growth was evaluated by counting the viable cell number with Cellomics Array-ScanTM VTI HCS Reader (Thermo Scientific, Waltham, MA, USA) according to the manufacturer's instructions. Briefly, HS-SY-II cells at 10 days post-transfection with candidate genes siRNA or NC-siRNA lentivirus were seeded at 2000 cells per well on 96-well plates, and then incubated at 37°C with 5% CO_2_. From the second day, cells with GFP were taken photos and counted each day by Cellomics machine. Cell growth was observed continuously for 5 days, and cell growth curves were drawn.

### MTT cell viability assay

MTT (3-(4, 5-dimethylthiazol-2-yl)-2, 5-diphenyltetrazolium bromide) cell viability assay was determined as previously described [14]. Briefly, cells were seeded in triplicate in 96-well plates at a density of 2 × 10^3^ cells/well in 100 μl culture medium. On the following day 1, 2, 3, 4 and 5, to evaluate the cell number, 10 μl MTT (5 mg/ml in PBS) (Sigma-Aldrich) was added to each well and the cells were incubated at 37°C in a humidified atmosphere containing 5% CO_2_. After 4 h, the medium was removed, and 100 μl dimethyl sulfoxide (DMSO, Sigma-Aldrich) was added to each well to terminate the reaction, and absorbency was measured at 490 nm with an EL-311SX enzymelinked immunosorbent assay reader (Bio-Tek Instruments, Winooski, Vermont, USA). This experiment was replicated three times.

### BrdU incorporation assay

Cells were seeded in triplicate in 96-well plates at a density of 2 × 10^3^ cells/well. A 5-bromo-2-deoxyuridine (BrdU) incorporation assay was detected on 1 and 4 days after seeding using the BrdU Cell Proliferation ELISA kit (Roche, USA) as previously described [43]. Briefly, 10 ml of 1/100 diluted BrdU was added to each well. After incubation for 8 h, the medium was carefully aspirated out and FixDenat solution (200 μl/well) was added into each well. Cells were then incubated at room temperature in the dark for 30 min, blocked in 5% bovine serum albumin (BSA) at room temperature in the dark for a further 30 min. Then 100 μl/well diluted Anti-BrdU-POD working solution (1:100) was added into each well, and incubated at room temperature for 90 min. The plates were washed three times, and then 100 μl substrate solution was added into each well. The cells were incubated at room temperature for 5 to 30 min until the solution was a deep blue, 50 μl 10% H_2_SO_4_ was added into each well, and the plates were read at 450 nm in an ELISA reader (Biotek Elx800, USA).

### Colony formation assay

Cells were plated at 500 cells per well in 6-well plates and continually cultured in DMEM containing 10% FBS for 10 days. Then, the supernatants were discarded and cells were washed twice with PBS, and fixed with methanol for 15 min. The cells were stained with 0.1% crystal violet for 10 min, subsequently, washed with PBS till the plates were clear. The plates were dried at room temperature and the colony numbers were photographed and counted. The experiments were performed in triplicate.

### Flow cytometry analysis of the cell cycle and apoptosis

Flow cytometry was used to analyze the effect of SHCBP1 on cell cycle. Cells were cultured in 6-well plates and were harvested by trypsinization and centrifugation at 1200 rmp for 5 min, washed once with ice cold PBS, and fixed in 70% alcohol for 1 h. Then, fixed cells were resuspended in PBS containing RNase (100 μg/ml) on ice, and stained with propidium iodide (PI, 50 μg/ml) (Sigma-Aldrich). Cells were analyzed using a FACScan flow cytometer (Becton Dickinson, San Jose, CA), according to the manufacturer's protocol. For Lentivirus-mediated RNAi knockdown of SHCBP1 experiments, cell apoptosis was determined by staining with Annexin V-APC Apoptosis Detection Kit (eBioscience, San Diego, CA, USA) and detected by FACS. For cells with overexpression of SHCBP1, cell apoptosis was assayed by staining with Annexin V-FITC (ebioscience) and PI following manufacturer's instructions and detected by FACS. Briefly, cells were collected and washed with cold PBS. They were then resuspended in 1 ml 1 × staining buffer. Then 5 μl Annexin V-APC and 5 ul PI was added into 100 μl of the above cell suspension (about 1 × 10^6^−1 × 10^7^ cells), and incubated for 15 min at room temperature in the dark. After incubation, cells were analyzed by flow cytometer in one hour. All experiments were repeated three times.

### SHCBP1 knock-down in a nude mouse tumor model

For the SS xenograft model, 4-week-old BALB/c nude mice (Vitalriver, Beijing, China) were housed in a temperature-controlled, pathogen-free environment and used for experimentation. Medium (200 μl) containing 2 × 10^6^ HS-SY-II cells at the end of SHCBP1-siRNA or NC-siRNA lentivirus infection for 48 h was injected subcutaneously into the right flank of nude mice. Tumor length (L), width (W) and diameter were measured every week from week 1 to week 6; tumor volume (mm^3^) was calculated using the formula L × W^2^/2 [44]. After 6 week, mice were sacrificed, and tumors were harvested.

### Statistical analysis

The data shown are presented as the mean ± standard deviation (SD) of three independent experiments. Statistical significance was determined with Student's *t* test using GraphPad Prism 5 software (GraphPad Software Inc., San Diego CA, USA). *P* < 0.05 was considered statistically significant.

## SUPPLEMENTARY MATERIAL FIGURES AND TABLE


