# Supplementary material for: Identification of SHCBP1 as a novel downstream target gene of SS18-SSX1 and its functional analysis in progression of synovial sarcoma
Source: Oncotarget. 2016 Aug 27;7(41):66822–34. doi: 10.18632/oncotarget.11651 (PMC5341840; doi:10.18632/oncotarget.11651)
Supplement: Supplementary file 1 [file oncotarget-07-66822-s001.pdf]

Identification of SHCBP1 as a novel downstream target gene of SS18-SSX1 and its functional analysis in progression of synovial sarcoma

Supplementary Materials

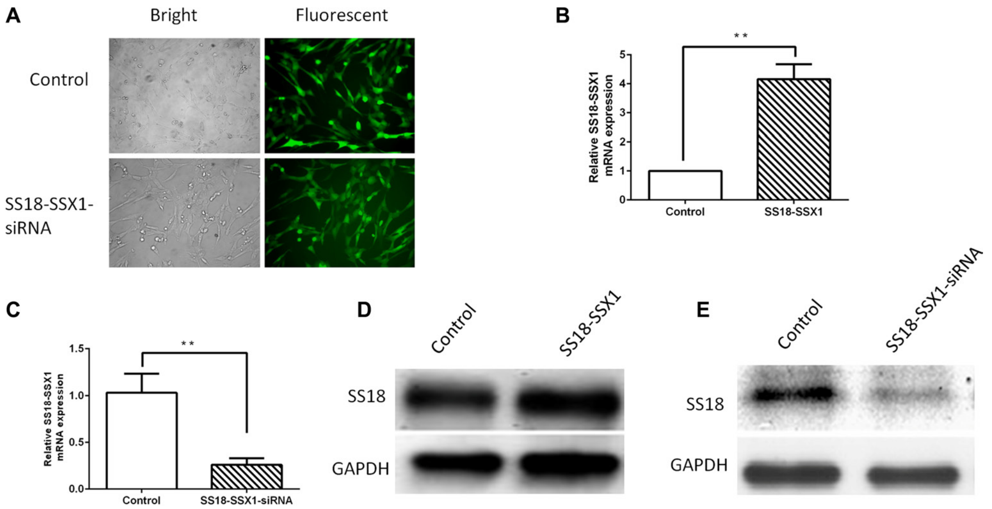

Supplementary Figure S1: qPCR and western blot analysis of SS18-SSX1 expression in SS18-SSX1-overexpressing and SS18-SSX1-silenced cells. (**A**) The transduction efficiency was determined at 3 days after infection with lentiviruses at a multiplicity of infection of 50. The transduced cells labeled with GFP were observed under a fluorescence microscope. Bright micrograph (left); fluorescent micrograph (right) (× 100). (**B** and **C**) qPCR analysis of SS18-SSX1 mRNA expression in SS18-SSX1-overexpressing (B) and SS18-SSX1-silenced (C) cells. Expression levels were normalized to GAPDH. Values are mean ± SD of three independent experiments; $^{**}p < 0.01$. (**D** and **E**) Western blot analysis of SS18-SSX1 in SS18-SSX1-overexpressing (D) and SS18-SSX1-silenced (E) cells. GAPDH was used as a loading control.

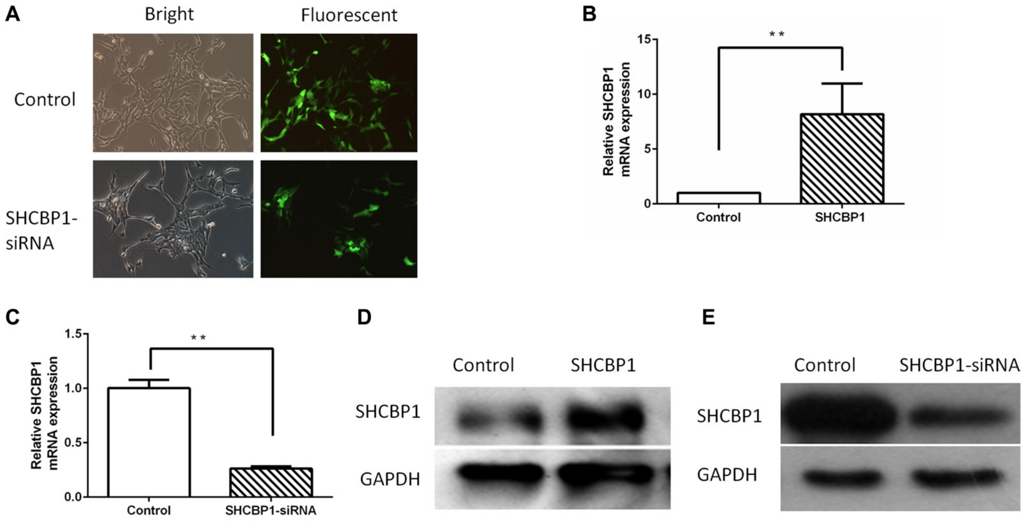

Supplementary Figure S2: qPCR and western blot analysis of SHCBP1 expression in SHCBP1-overexpressing and SHCBP1-silenced cells. (**A**) Cells infected with lentivirus at a multiplicity of infection of 50 for 72 h. Bright (left) and fluorescent (right) photomicrographs of HS-SY-II cells were taken after lentivirus infection at a magnification of 100. (**B** and **C**) qPCR analysis of SHCBP1 mRNA expression in SHCBP1-overexpressing (B) and SHCBP1-silenced (C) cells. Expression levels were normalized to GAPDH. Values are mean ± SD of three independent experiments; $^{**}p < 0.01$. (**D** and **E**) Western blot analysis of SHCBP1 in SHCBP1-overexpressing (D) and SHCBP1-silenced (E) cells. GAPDH was used as a loading control.

Supplementary Table S1:The primers applied in the study

Description	Name	Sequence
Primers for qPCR	SS18-SSX1-F	GGACAACCAATGGGAAAC
	SS18-SSX1-R	TTGTATTGCTGAGAAGGAGG
	SHCBP1-F	GCTACCGTGATAAACCAGGTTC
	SHCBP1-R	AGGCTCTGAATCGCTCATAGA
	Nidogen 2-F	TAGGCGCTTACGAGGAGGTCAA
	Nidogen 2-R	TATCAGACCCATCAGATGCCAAAAC
	HOXC11-F	GTGAAGGGAAGTGTCTGATGCA
	HOXC11-R	AATCCGAGCAGCAAGACATTG
	GAPDH-F	TGACTTCAACAGCGACACCCA
	GAPDH-R	CACCCTGTTGCTGTAGCCAAA
Primers for vectors construction	pcDNA .1(+)-SS18-SSX1-F	GGAATTCATGTCTGTGGCTTTCGCGG
	pcDNA .1(+)-SS18-SSX1-R	GGCGAGCTCTTACTCGTCATCTTCCTCAGGGTC
	pcDNA .1(+)-SHCBP1-F	GGAATTCATGGCTGACGGGTCGCTGAC
	pcDNA .1(+)-SHCBP1-R	GGCGAGCTCTCAGAAAAGAAATGTGCCA
